# Robust Depth Estimation and Image Fusion Based on Optimal Area Selection

**DOI:** 10.3390/s130911636

**Published:** 2013-09-04

**Authors:** Ik-Hyun Lee, Muhammad Tariq Mahmood, Tae-Sun Choi

**Affiliations:** 1 School of Mechatronics, Gwangju Institute of Science and Technology (GIST), 123 Cheomdan gwagiro, Buk-Gu, Gwangju 500-712, Korea; E-Mail: ihlee@gist.ac.kr; 2 School of Computer Science and Engineering, Korea University of Technology and Education, 1600 Chungjeolno, Byeogchunmyun, Cheonan, Chungnam 330-708, Korea; E-Mail: tariq@koreatech.ac.kr

**Keywords:** depth estimation, optimal area selection, 3D camera

## Abstract

Mostly, 3D cameras having depth sensing capabilities employ active depth estimation techniques, such as stereo, the triangulation method or time-of-flight. However, these methods are expensive. The cost can be reduced by applying optical passive methods, as they are inexpensive and efficient. In this paper, we suggest the use of one of the passive optical methods named shape from focus (SFF) for 3D cameras. In the proposed scheme, first, an adaptive window is computed through an iterative process using a criterion. Then, the window is divided into four regions. In the next step, the best focused area among the four regions is selected based on variation in the data. The effectiveness of the proposed scheme is validated using image sequences of synthetic and real objects. Comparative analysis based on statistical metrics correlation, mean square error (MSE), universal image quality index (UIQI) and structural similarity (SSIM) shows the effectiveness of the proposed scheme.

## 1. Introduction

Depth information of an object is very useful and advantageous in many computer vision applications. Therefore, 3D cameras with depth sensing capabilities are becoming more popular and have a wide range of applications in the consumer electronics community. Web-conferencing, 3D gaming, objects tracking, face detection and tracking, automotive safety, mobile phones, robotics and medical devices are potential areas that are using depth cameras with a high expense. These cameras compute depth using various techniques, such as time of flight, stereo or triangulation and monocular [1]. In general, 3D camera systems are expensive and complex [2]. Alternately, optical passive methods can be a good solution. These methods are inexpensive and fast. However, accurate and robust depth estimation of an object through optical methods is a challenging task.

Shape from focus (SFF) is one of the optical methods used to recover the shape of an object from a stack of monochrome images [3–5]. In this technique, a sequence of images is acquired at different focus levels by translating the object along the optical axis. Imaging devices, particularly those with lenses of long focal lengths, usually suffer from limited depth-of-field. Therefore, in the acquired images, some parts of the object are well-focused, while the other parts are defocused, with a degree of blur. In the SFF technique, in the first step, a focus measure is applied to compute sharpness or quality of focus for each pixel in the image sequence [6–9]. After applying a focus measure operator on the image sequence, an image focus volume is obtained. A rough depth map is then obtained by maximizing the focus measure along the optical axis. SFF methods are successfully utilized in various industrial applications, such as surface roughness measurement and focus variation for area-based 3D surface measurement [10]. Further, in the case of dynamic scenes, SFF can be used to reconstruct 3D shape by using telecentric optics [11]. In the second step, an approximation technique is used to further refine the initial depth map [5,12,13]. The performance of these techniques depends on the accuracies of the focus measures. Focus measures usually suffer from inaccuracies in focus quality assessment. In order to enhance the initial focus volume, usually, all focus values within a fixed window are aggregated [3,14]. However, this summation does not provide an accurate depth map [15–17]. It causes the over-smoothness of the object shape and, more likely, removes the edges. Particularly, in a noisy environment, its performance is deteriorated.

In this paper, we introduce the optimal computing area for robust focus measurement in SFF. Although the fixed small window provides a good depth map, there remains notable inaccuracies in recovered 3D shapes. In the proposed scheme, first, an adaptive window is computed through an iterative process using a criterion. Then, the window is divided into four regions. Each region contains the central pixel. In the next step, the best focused area is selected based on variation in the data. The effectiveness of the proposed scheme is validated using image sequences of synthetic and real objects. Comparative analysis based on statistical metrics correlation, mean square error (MSE), universal image quality index (UIQI) [18] and structural similarity (SSIM) [19] shows the effectiveness of the proposed scheme.

## 2. Background

**Figure 1.**
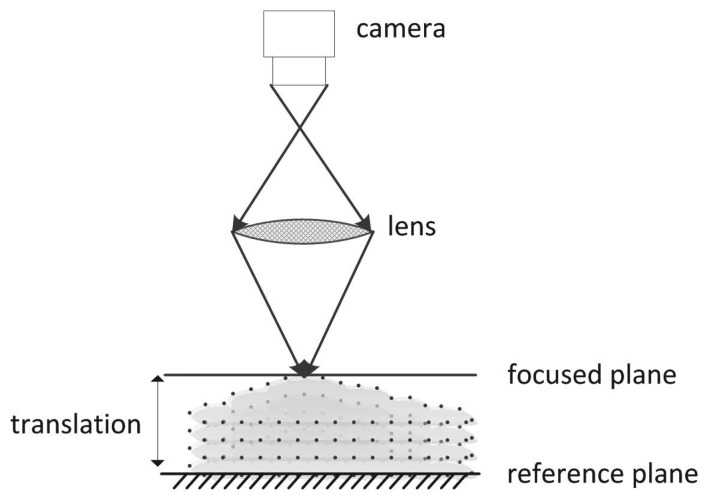
Basic schematic of shape from focus (SFF).

In SFF, the objective is to find out the depth by measuring the distance of a well-focused position of every object point from the camera lens. Once distances for all points of the object are found, the 3D shape can easily be recovered. Figure 1 shows the basic schematic of SFF. Initially, an object of unknown depth is kept on a reference plane and, then, translated in the optical direction in fixed finite steps with respect to a real aperture camera. At every step, an image is captured, and a stack of visual observations are obtained. Due to the limited depth-of-field of the camera and the 3D nature of the object, the captured images are space-variantly blurred, such that some parts of the object come into focus in each frame. The distances between the focus plane and reference plane are known. Measuring the true focus point requires a large number of images with incremental distance movement towards the focus plane.

In the literature, many SFF techniques have been proposed. Usually, the SFF method consists of two major parts. First, a focus measure is applied to measure the focus quality of each pixel in the image sequence, and an initial depth is computed by maximizing the focus measure in the optical direction. Second, an approximation technique is applied to enhance the initial depth. In order to detect the true focus point from a finite number of images, a focus measure, a criterion to measure the focus quality, is applied. A focus measure is a quantity that measures the degree of blurring of an image; its value is a maximum when the image is best focused and decreases as blurring increases. In the literature, many focus measures have been proposed in spatial and frequency domains. One of the famous categories of focus measures in the spatial domain is based on image derivatives. These focus measures are based on the idea that the larger difference in intensity values of neighboring pixels are analogous to sharper edges. Broadly, they can be divided into two sub-categories: first and second derivative-based methods. A method based on gradient energy is investigated by Tenenbaum [20]. The Tenenbaum function (TEN) is a gradient magnitude maximization method that uses the Sobel operators to estimate the gradient of the image. Several focus measures have been proposed by modifying the Laplacian (ML) operator [3]. Among these, the sum of the modified Laplacian (SML) focus measure based on the second derivative has gained considerable attention [3]. In this focus measure, first, an image is convolved with the Laplacian operator; then, it is modified by taking the energy of the Laplacian. In order to improve the robustness for a weak textured image, the resultant values are summed up within a small window. Many focus measures have been reported based on the statistical analysis of image intensities [9,21]. Intuitively, high variance is associated with sharp image structure, while low variance is associated with blurring, which reduces the amount of gray-level fluctuation. The larger variance of intensity values within a small window corresponds to a sharper image and *vice versa*. This method is called gray level variance (GLV) [22,23].

Some focus measures have also been proposed in the transform domain. Kristan *et al.* [24] proposed another focus measure by using Bayes spectral entropy function. Baina and Dublet [25] proposed the energy of the alternative current (AC) part of discrete cosine transform (DCT) as a focus measure. Kubota and Aizawa [26] proposed two focus measures in the wavelet domain. These focus measures are very similar to the first and second order moments of the high frequency components. Xie *et al.* [27] proposed another focus measure in the wavelet domain. The ratio of the energies of the high frequency components to the low frequency components is taken as a focus quality measure.

Once an initial depth estimate is obtained by applying a focus measure, a refinement procedure is followed to further refine the results. In the literature, various approximation-and machine learning-based refinement techniques have been proposed [3,8,28,29]. Some approaches use interpolation techniques for surface approximation [8,28]. However, fitting of image focus curves to Gaussian or any other model may not provide the optimal depth, as focus curves do not always follow the specific model. Additionally, the initial estimated depth map contains errors, due to the inaccuracies of the focus measure. This is because the initial focus measure may enhance noise related to intensity variation instead of actual intensity variation. On the other hand, machine learning-based approaches provide better results, as compared to interpolation techniques [8,22]. However, they also suffer from a generalization problem. The learned models may not provide optimal results for images of diverse objects taken under diverse conditions.

## 3. Proposed Method

An image sequence, *I_z_*(*x*, *y*), consisting of *Z* images, each of size *X* × *Y* , is obtained through a charge-coupled device (CCD) camera by translating the object in small steps along the optical axis. The focus quality of each pixel in the sequence is determined by applying a focus measure locally. For each pixel, (*x*, *y*), in the image sequence, the window, *R*(*x*, *y*), of the size, *M* × *N*, is used to compute the focus measure, *i.e.*,

(1) $$R(x,y)=\left\{(\xi ,\zeta )||\xi -x|\leq \frac{J}{2}\wedge \left|\zeta -y\right|\leq \frac{K}{2}\right\}.$$

where *J* and *K* determine the size of the window. In the case of a square image patch, *J* is equal to *K*.

**Figure 2.**
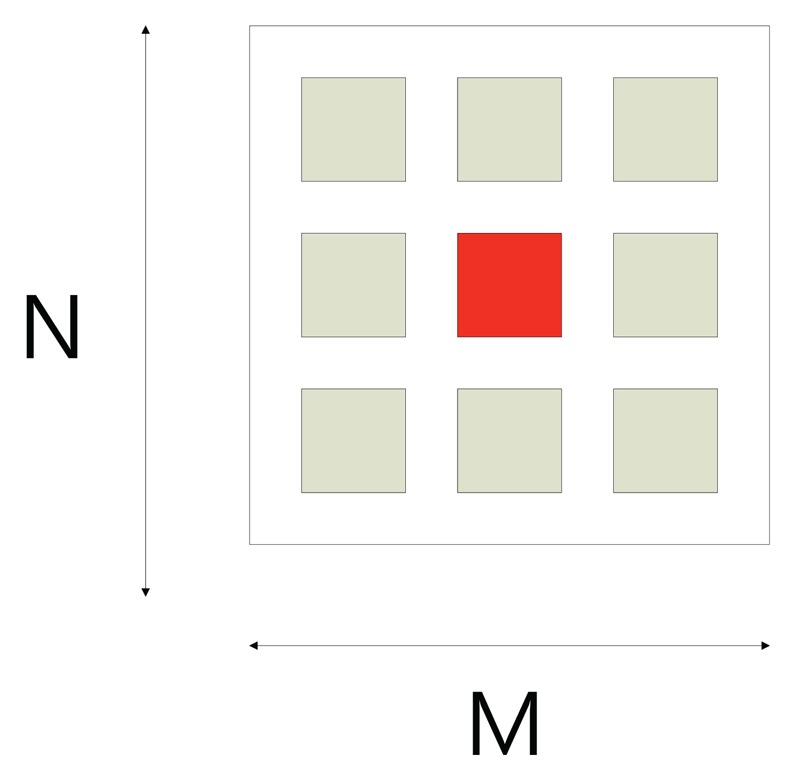
Conventional computing area.

Figure 2 shows the conventional eight neighborhood pixels around the central pixel. Once we have obtained a window of appropriate size that contains sufficient data variation, the next step is to compute the focus measure. Conventionally, the pixels in the whole window have been used to compute focus quality. However, the computed focus measure may not be robust. Usually, noise in the image is also related with a high frequency component. As the focus measure computes focus quality by computing the high frequency components (high pass filter), so there are chances that noise-related intensities may also contribute to the focus measure. To eliminate this factor, we propose to divide the region into parts, and then, the focus measure is computed from the part that maximizes the focus measure. Each region is slightly overlapped with others and contains the central pixel of the window. In the first step, we set the input image patch of size *J* × *K* around central pixel point, (*x*, *y*), *J* = *K* = 4*L* + 1, where *L* is an integer. In the next step, the input image patch is divided into four regions, *R_i_*, *i* = 1 ⋯ 4, each of size *M* × *N*. The sizes of sub-windows are related as follows:

(2) M×N=[2L+1]×[2L+1].

**Figure 3.**
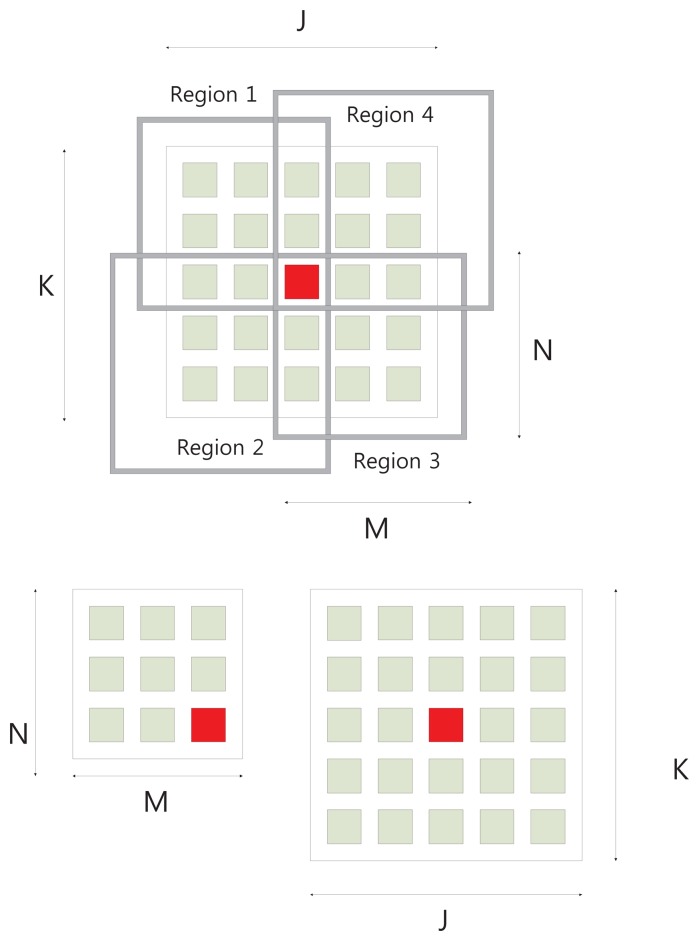
Proposed computing area.

Figure 3 shows the input image patch and four sub-windows around the center pixel. The proposed focus measure is computed by selecting one of the sub-window. In order to select the optimal computing area, we calculate mean (*μ*) and variance (*σ*^2^) for each region.

(3) $$\mu_{i}=\frac{1}{\text{MN}}\sum R_{i}(x,y)\;,i=1,\ldots ,4.$$

(4) $$\sigma_{i}^{2}=\frac{1}{\text{MN}-1}\sum {\left(R_{i}(x,y)-\mu_{i}\right)}^{2}\;,i=1,\ldots ,4.$$

The optimal computing area is selected depending on the variance within the four regions. We choose the area having the maximum variance among all four regions. Thus, the area within the window is selected as:

(5) $$R_{i}(x,y)=\{R_{i}:\sigma_{i}^{2}=\max\{\sigma_{i}^{2}|i=1,\ldots ,4.\}\}.$$

The high variance depicts high contrast or high frequency. Therefore, the value of the focus measure increases as contrast increases, and this affects the maximum sharpest focused image. By applying the focus measure on each pixel of the image sequence, an initial focus volume, *F_z_*(*x*, *y*), is obtained as:

(6) $$F_{z}(x,y)=\text{FM}\;(I_{z}\;(x,y)).$$

where *I_z_* (*x*, *y*) is an image sequence, *FM* indicates a focus measure, such as GLV [22,23], SML [3] or TEN [20], and *F_z_* (*x*, *y*) is the focus volume obtained after applying *FM* on the input image sequence.

**Figure 4.**
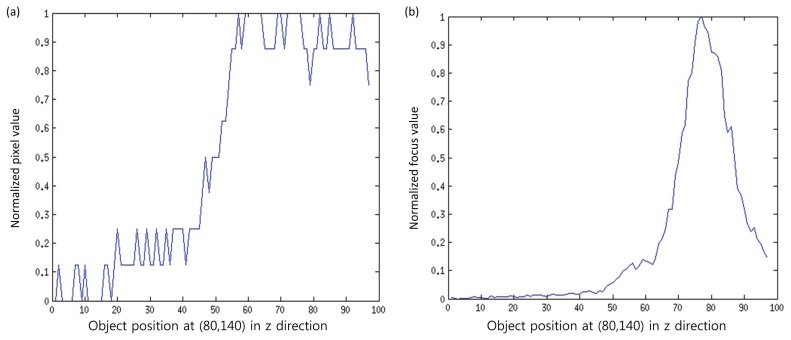
Curves for pixels at (40, 180) of a real cone image sequence: (**a**) original gray level values; (**b**) after applying the proposed focus measure.

It is notable that noise in the image is usually related with high frequency components. As the focus measure computes the focus quality by computing the high frequency components (high pass filter), so there are chances that noise-related intensities may also contribute to the focus measure. To eliminate this factor, we propose to divide the input patch into sub-windows, and then, the focus measure is computed from the part that maximizes the focus measure. Figure 4 shows the effectiveness of the proposed focus measure. In this figure, curves for the original signal and the signal obtained by the proposed method for pixels at (80, 140) of a real cone are shown. It can be observed that the original signal contains noise, while the processed signal is smooth and has a clear, single peak. This peak (maximum focus measure) indicates the depth for the pixel (80,140). In this way, the entire depth map, *E_D_*(*x*, *y*), is calculated by using the best focused points in the focus volume, *F_z_*(*x*, *y*), as follows:

(7) $$E_{D}\;(x,y)=\underset{z}{\arg\max}F_{z}(x,y).$$

The best focused values provide an image of better quality of the object [30] that is focused everywhere. Therefore, *FI* (Focused Image) is computed from the image focus volume as, *i.e.*:

(8) $$\text{FI}\;(x,y)=I_{E_{D}(x,y)}(x,y).$$

**Figure 5.**
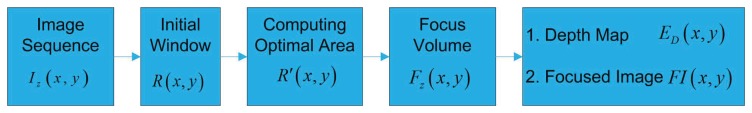
The diagram of the proposed method.

The complete procedure of the proposed method is illustrated in Figure 5. The summary of computing the optimal area is presented in Algorithm 1.


**Algorithm 1** Determination of the optimal area.
1:**procedure** Optimal computing area(OCA)2:*R*(*x*, *y*)▹ Initial window, *R*(*x*, *y*), with size *J*, *K*3:*M* = *N* ← [2*L* + 1] × [2*L* + 1]▹ Four regions, *R_i_*, *i* = 1, 2, ⋯, 4.4:*μ_i_*▹ Calculation of each regions' mean5:*σ_i_*▹ Calculation of each regions' variance6:
$\text{OCA}\leftarrow \{R_{i}:\sigma_{i}^{2}=\max\{\sigma_{i}^{2}|i=1,\cdots ,4.\}\}$▹ Finding of the maximum variance area for the optimal area selection7:**end procedure**


## 4. Results and Discussions

### 4.1. Test Images

#### 4.1.1. Synthetic Images

**Figure 6.**
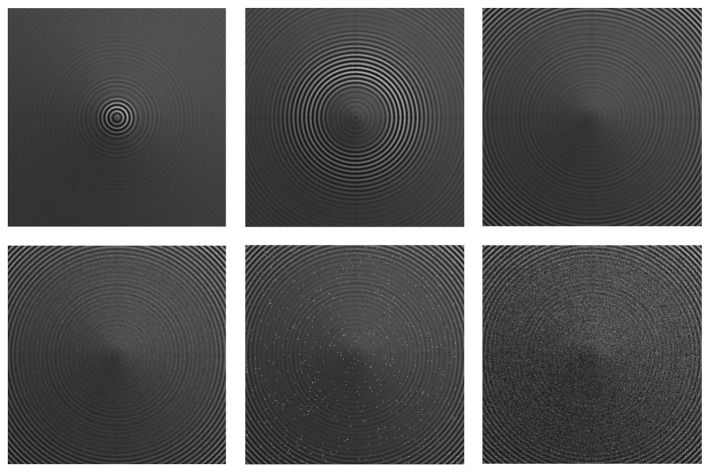
Sample images from the sequence of a simulated cone: noise-free (first row), in the presence of noise (second row), Gaussian noise with zero mean and a variance of 0.01 (first column), salt and pepper noise with a variance of 0.01 (second column) and speckle noise with a variance of 0.01 (last column).

The images for a simulated cone object were generated using camera simulation software. The simulated cone has been selected for the experiments, because it is easy to verify the results for such an object with a known data depth map. Images of the simulated cone at different lens positions are shown in Figure 6. From the images, we see that some parts of the cone are focused at one lens position, while other parts of the cone are focused at other lens positions. Our target is to get all focused parts and to reconstruct the 3D shape of the cone. The dimension of image sequence *I_z_*(*x*, *y*) is 360 × 360 × 97. More details about the procedure and image generator can be found in [31].

#### 4.1.2. Real Images

**Figure 7.**
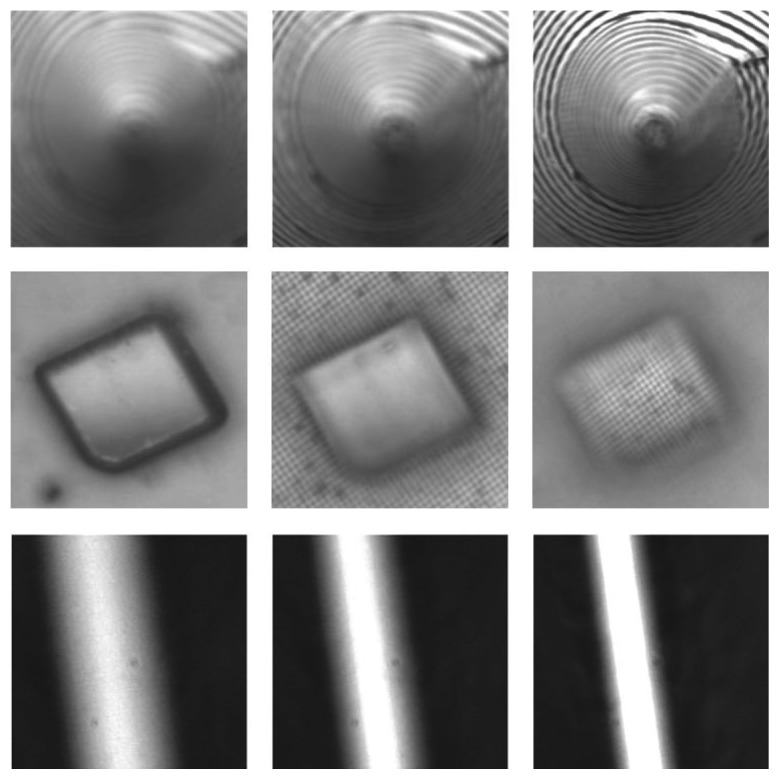
Sample images from the sequence of real objects: real cone (first row), micro sheet (second row), groove (bottom).

In order to investigate the performance of different focus measures and SFF techniques in real scenarios, several experiments have been conducted using an image sequence of real objects. A sequence of 97 images of a real cone object, each of 200 × 200 dimensions, has also been used in many experiments. The real cone object is made of hardboard with black and white strips drawn on its surface to enrich the texture. The length of the cone is about 97 inches, while the base diameter is about 15 inches. Details of these test images can be found in [4]. Another sample was a micro sheet constructed by preparing copper solution through *Cu*(*NO*_3_)_2_3*H*_2_*O*, *NaOH* and distilled water. Under specific temperature, the solution was then transferred into Teflon-lined stainless steel autoclave of 100 mL capacity for a certain time. For the third sample, the images for the real object groove consisted of 60 images, each of a size of 300 × 300 dimensions. Figure 7 shows the sample images of real objects.

### 4.2. Performance Comparison Metrics

For performance assessment and evaluation, we used two statistical metrics: mean square error (MSE) and correlation (C2). The lower value of the MSE indicates that the method provides more accuracy and higher precision. The correlation value provides the similarity between the real and estimated depth map. The higher the correlation is, the closer it is to the original image. This means that the depth map is well estimated. Recently, new metrics for comparison were developed by Zhou and Bovik. The Universal Image Quality Index (UIQI) [18] is a quality index that models image distortion by combining three factors: loss of correlation, luminance distortion and contrast distortion. The dynamic range of UIQI is [−1, 1]. One is the best value for comparison. An extension of UIQI is also suggested by Zhou and Bovik. The Structural Similarity (SSIM) index [19] measures the similarity between two images.

Contrary to simulated objects, it is to obtain depth information for real objects. Although real objects cannot use statistical metrics, other metrics can be used, such as surface smoothness [32]. The surface smoothness is used for comparison of the conventional and proposed methods. A higher *SS* (surface smoothness) value implies that the surface is smoother. Table 1 depicts the best or ideal value of the output value after computing the difference between actual depth and estimated depth.

### 4.3. Focus Measures Comparison

**Figure 8.**
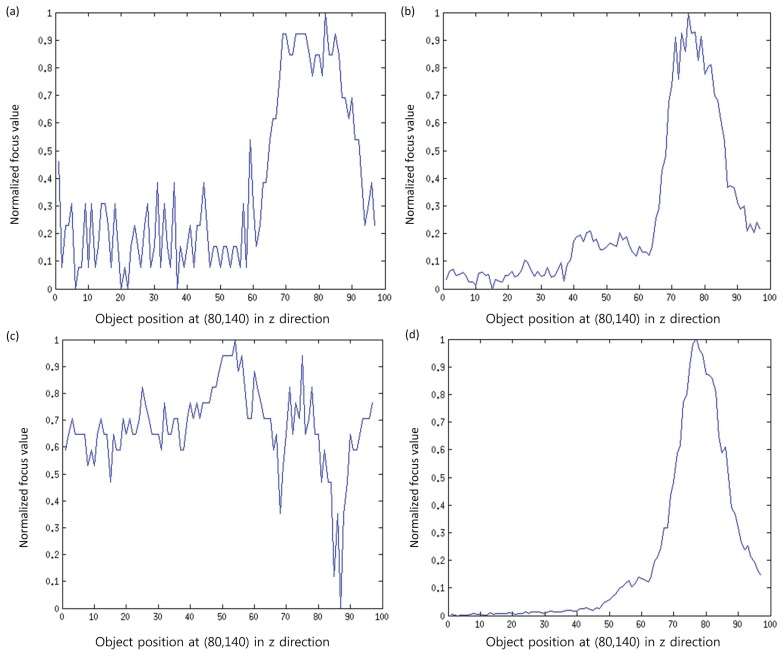
Focus curves by applying (**a**) modified Laplacian (ML); (**b**) gray level variance (GLV); (**c**) Tenenbaum function (TEN); (**d**) optimal computing area (OCA).

In order to investigate the improved performance of the proposed method, the results are compared with the traditional methods, such as SML, GLV and TEN. In our experiments, we set *J* = *K* = 5 based on an analysis done by Malik and Choi [33] for window size selection. Thus, the size of the sub-window is *M* × *N* = 3 × 3. The proposed method for computing a sub-window is simple; however, if some sophisticated technique is applied to select the noise-free pixels from the initial window, then more accurate results are expected. In addition, the proposed method is better than simply computing the focus measure using a smaller window, as in the proposed method, some noisy parts are not taking part while computing the focus measure. Some intermediate results are presented in Figure 8. It can be observed that the focus curves obtained by the proposed method are smoother compared to the curves obtained through the other methods.

### 4.4. Experiment with Synthetic Images

#### 4.4.1. Noise-Free Condition

**Figure 9.**
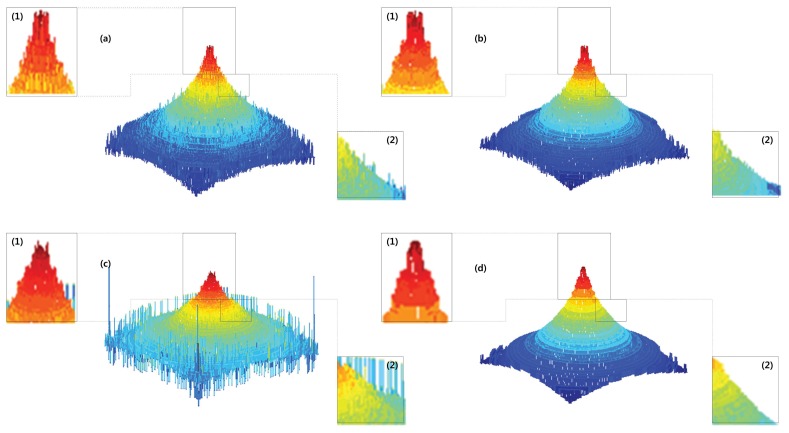
Depth maps for simulated cone: (**a**) ML; (**b**) GLV; (c) TEN; (**d**) the proposed method and their (1) tip; (2) side.

Figure 9 shows the comparison of the results obtained from different focus measure methods using the image sequence of the simulated cone. To distinguish the shape difference can be difficult but it is easy to see the tip and some parts of the cone.

Table 2 shows the comparison for conventional focus measures and the proposed optimal computing area-based method (OCA). The proposed method has the lowest MSE value among the others. It depicts that the proposed method is more accurate and has higher precision. The highest value of the correlation is also obtained through the proposed method. This means that the depth map is well estimated. In addition, compared to two other metrics—UIQI and SSIM—the proposed method has produced the highest values among them.

#### 4.4.2. Various Noise Condition

**Figure 10.**
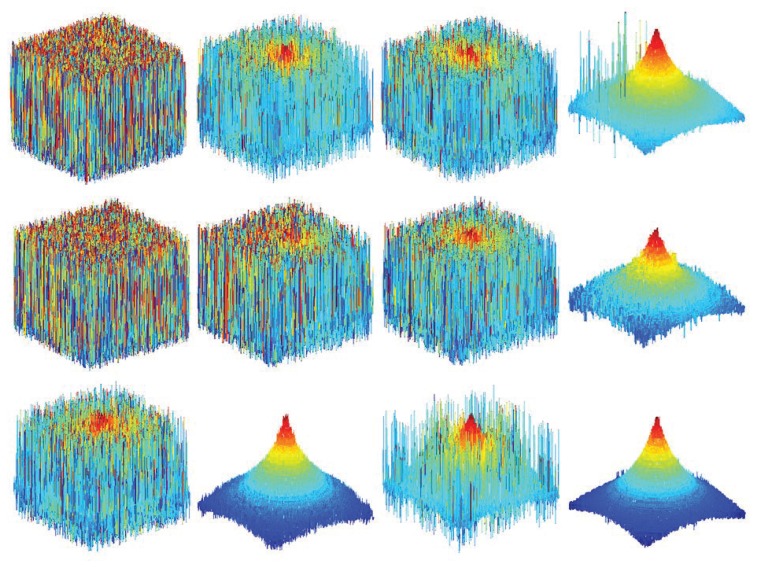
Depth maps: Gaussian noise with zero mean and a variance of 0.01 (first row), salt and pepper noise with a variance of 0.01 (second row), speckle noise with a variance pf 0.01 (bottom), ML (first column), GLV (second column), TEN (third column) and the proposed method (last column).

We deal with various noise type such as Gaussian, salt& pepper, speckle noise. Figure 10 (second, last row) show the reconstructed 3D shape in presence of salt& pepper, speckle respectively. The proposed methods provide strong denoised 3D shape then other conventional method.

Table 3 shows the comparison of various SFF methods for the robustness in the presence of Gaussian noise with zero mean and 0.01 variance and the salt and pepper noise with 0.01 density. It can be observed that the proposed method has shown better performance compared to the conventional methods.

**Figure 11.**
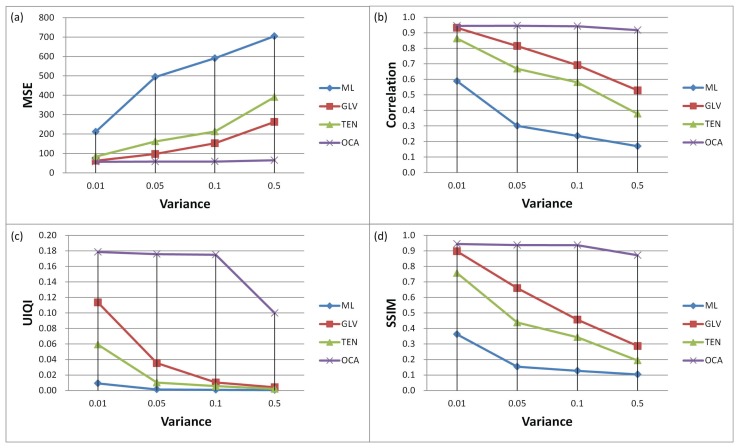
Comparison of SFF methods for various speckle noise variances.

Further, we have conducted simulations by using an image sequence corrupted with speckle noise with different noise variances. Figure 11 shows the qualitative measures for different SFF methods. It can be observed that in the presence of noise, the proposed method has provided the best performance among the others. The MSE and the correlation values for the proposed method are stable compared to the conventional approaches. The performance of the conventional methods is degraded quickly with the increase of noise levels, whereas the proposed method has shown considerable resistance against noise.

In addition, the overall rank of each method can be seen in Table 4. Each method shows different robustness against various noises. GLV is second in the presence of Gaussian and speckle, except salt and pepper noise. TEN has the second best performance against salt and pepper noise. The proposed method shows the best performance among the others. The added noise results of the proposed method are almost the same as the no noise results.

### 4.5. Experiment with Real Images

**Figure 12.**
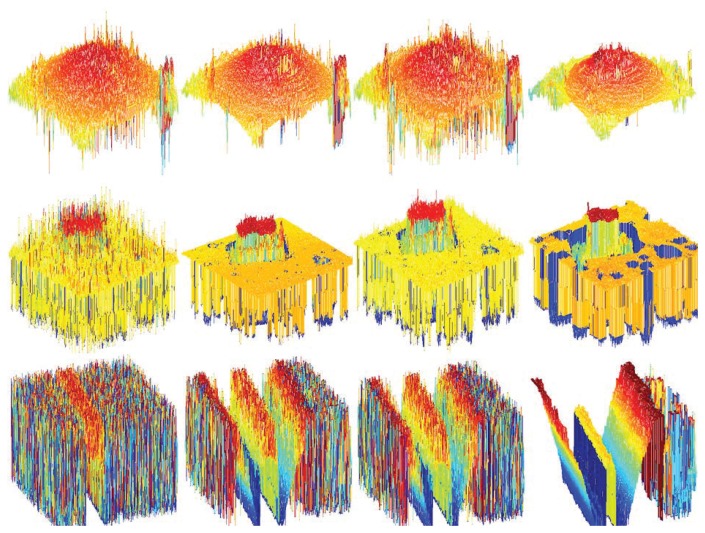
Depth maps: real cone (first row), micro sheet (second row), groove (bottom), ML (first column), GLV (second column), TEN (third column) and the proposed method (last column).

The surface of the object is a key point for comparison. The smooth surface of the planar object can be seen in the proposed method. The reconstructed real cone 3D shape is in Figure 12 (first row). Except one peak in the middle of the object surface, the proposed method is better than the other methods. Figure 12 (second row) shows the reconstructed 3D of the micro sheet. The proposed methods providing more noise reduced the shape compared to the others. The shape of groove object image is shown in Figure 12 (last row).

Table 5 shows the reconstructed object surface smoothness. The proposed methods provide a smoother surface compared to conventional methods.

In addition, the overall rank of each method can be seen in Table 6. GLV ranks second with various real objects. The proposed method shows the best performance among the others.

**Figure 13.**
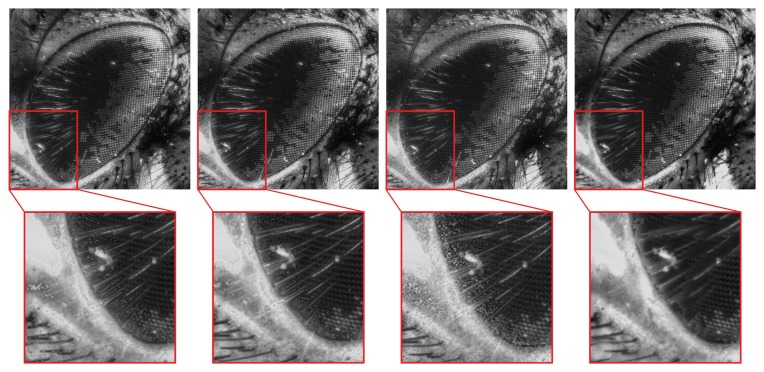
Fused images using: ML (first column), GLV (second column), TEN (third column) and OCA (last column).

Figure 13 shows the fused images using conventional methods and the proposed method. The second column figures show the magnified partial images of the images shown in red boxes. The proposed method has provided better quality and a less noisy image compared to other conventional methods.

In the literature [34,35], researchers used denoising filtering, both pre-processing and post-processing, to remove possible noise caused by the sensor or the initial depth estimation. However, the use of denoising techniques before computing the focus measure is not so effective, as these techniques will also remove the edges and effect the sharpness of the image, which will result in inaccurate computation of the focus measurements.

## 5. Conclusions

In this paper, we introduced the optimal computing area of the area; the highest mean absolute derivation region is selected as the focus measure. The proposed algorithm has been exterminated using image sequences of a synthetic and various real objects: a micro sheet, a real cone and a groove. We performed experiments with image sequences corrupted with Gaussian, salt and pepper and speckle noise. From the experimental results, we can finalize the main properties of the proposed focus measure.

- Robustness: The proposed method has shown the robustness against various noise, even high noise variance (0.01) or noise density (0.01).
- Accuracy: For various qualitative measures, the proposed method has provided better results (94.47% similar to true depth) than conventional methods (92.28%–93.83% similar to true depth).

## Figures and Tables

**Table 1.** The best value of various metrics.

**Metrics**	**Description**	**Ideal Value**
MSE	Mean Square Error	Minimum
Corr	Correlation	1
UIQI	A Universal Image Quality Index	1
SSIM	The Structure Similarity Index	1
SS	Surface Smoothness	Minimum

**Table 2.** Comparison for SFF methods with various metrics.

**Metrics**	**ML**	**GLV**	**TEN**	**OCA**
MSE	58.6907	52.3340	57.0221	51.5491
Corr	0.9253	0.9383	0.9228	0.9447
UIQI	0.0795	0.1176	0.0924	0.1989
SSIM	0.8460	0.9021	0.8549	0.9533

**Table 3.** Comparison of SFF methods with Gaussian noise (zero mean and 0.01 variance) and salt and pepper noise (noise density 0.01).

**Metrics**	**Noise**	**ML**	**GLV**	**TEN**	**OCA**
MSE	Gaussian	695.4148	165.7734	217.7481	50.4545
	salt and pepper	830.5300	498.7118	324.2682	71.4485
	No noise	58.6907	52.3340	57.0221	51.5491

Corr	Gaussian	0.0641	0.5857	0.4926	0.9362
	salt and pepper	0.0866	0.2895	0.4476	0.9093
	No noise	0.9253	0.9383	0.9228	0.9447

UIQI	Gaussian	0.0002	0.0109	0.0047	0.1712
	salt and pepper	0.0002	0.0025	0.0039	0.0871
	No noise	0.0795	0.1176	0.0924	0.1989

SSIM	Gaussian	0.0825	0.3910	0.2672	0.9393
	salt & pepper	0.0864	0.1859	0.2392	0.8407
	No noise	0.8460	0.9021	0.8549	0.9533

**Table 4.** Overall rank table of each focus measure in the presence of various noise.

**Rank**	**Gaussian**	**Salt&Pepper**	**Speckle**
(1)	OCA	OCA	OCA
(2)	GLV	TEN	GLV
(3)	TEN	GLV	TEN
(4)	ML	ML	ML

**Table 5.** Surface smoothness comparison of various focus measures

**Objects**	**ML**	**GLV**	**TEN**	**OCA**
Simulated cone	193.4657	68.3371	170.6478	43.3136
Real cone	766.7228	227.8418	1,013.3	144.5903
Micro sheet	2,295.8	530.7807	942.2703	305.0307
Groove	4,517.9	1,267.3	1,790.3	252.9055

**Table 6.** Overall rank table of each focus measure with various objects.

**Rank**	**Simulated Cone**	**Real Cone**	**Micro Sheet**	**Groove**
(1)	OCA	OCA	OCA	OCA
(2)	GLV	GLV	GLV	GLV
(3)	TEN	ML	TEN	TEN
(4)	ML	TEN	ML	ML

## References

[1] Tran C., Trivedi M. (2011). 3D posture and gesture recognition for interactivity in smart spaces. IEEE Trans. Ind. Inform..

[2] Malik A., Choi T. (2007). Application of passive techniques for three dimensional cameras. IEEE Trans. Consum. Electron..

[3] Nayar S., Nakagawa Y. (1994). Shape from focus. IEEE Trans. Pattern Anal. Mach. Intell..

[4] Subbarao M., Choi T. (1995). Accurate recovery of three-dimensional shape from image focus. IEEE Trans. Pattern Anal. Mach. Intell..

[5] Sahay R., Rajagopalan A. (2011). Dealing with parallax in shape-from-focus. IEEE Trans. Image Process..

[6] Subbarao M., Tyan J. (1998). Selecting the optimal focus measure for autofocusing and depth-from-focus. IEEE Trans. Pattern Anal. Mach. Intell..

[7] Krotkov E. (1988). Focusing. Int. J. Comput. Vis..

[8] Zhang Y., Zhang Y., Wen C. (2000). A new focus measure method using moments. Image Vis. Comput..

[9] Wee C., Paramesran R. (2007). Measure of image sharpness using eigenvalues. Inf. Sci..

[10] Alicona Surface roughness measurement. http://www.alicona.com/home/en.

[11] Nayar S., Watanabe M., Noguchi M. (1996). Real-time focus range sensor. IEEE Trans. Pattern Anal. Mach. Intell..

[12] Ahmad M., Choi T. (2005). A heuristic approach for finding best focused shape. IEEE Trans. Circuits Sys. Video Tech..

[13] Pradeep K., Rajagopalan A. (2007). Improving shape from focus using defocus cue. IEEE Trans. Image Process..

[14] Thelen A., Frey S., Hirsch S., Hering P. (2009). Improvements in shape-from-focus for holographic reconstructions with regard to focus operators, neighborhood-size, and height value interpolation. IEEE Trans. Image Process..

[15] Mahmood M., Choi T. (2012). Nonlinear approach for enhancement of image focus volume in shape from focus. IEEE Trans. Image Process..

[16] Lee I.H., Shim S.O., Choi T.S. (2013). Improving focus measurement via variable window shape on surface radiance distribution for 3D shape reconstruction. Opt. Lasers Eng..

[17] Lee I., Tariq Mahmood M., Choi T.S. (2013). Adaptive window selection for 3D shape recovery from image focus. Opt. Laser Technol..

[18] Wang Z., Bovik A. (2002). A universal image quality index. IEEE Signal Process. Lett..

[19] Wang Z., Bovik A., Sheikh H., Simoncelli E. (2004). Image quality assessment: From error visibility to structural similarity. IEEE Trans. Image Process..

[20] Tenenbaum J. (1970). Accommodation in computer vision. Ph.D. Thesis.

[21] Xu X., Wang Y., Tang J., Zhang X., Liu X. (2011). Robust automatic focus algorithm for low contrast images using a new contrast measure. Sensors.

[22] Groen F., Young I., Ligthart G. (1985). A comparison of different focus functions for use in autofocus algorithms. Cytometry.

[23] Yeo T., Ong S., Sinniah R. (1993). Autofocusing for tissue microscopy. Image Vis. Comput..

[24] Kristan M., Pers J., Perse M., Kovacic S. (2006). A Bayes-spectral-entropy-based measure of camera focus using a discrete cosine transform. Pattern Recognit. Lett..

[25] Baina J., Dublet J. Automatic focus and Iris Control for Video Cameras.

[26] Kubota A., Aizawa K. (2005). Reconstructing arbitrarily focused images from two differently focused images using linear filters. IEEE Trans. Image Process..

[27] Xie H., Rong W., Sun L. (2007). Construction and evaluation of a wavelet-based focus measure for microscopy imaging. Microsc. Res. Tech..

[28] Favaro P., Soatto S., Burger M., Osher S. (2008). Shape from defocus via diffusion. IEEE Trans. Pattern Anal. Mach. Intell..

[29] Subbarao M., Choi T., Nikzad A. (1993). Focusing techniques. Opt. Eng..

[30] Luo R., Yih C., Su K. (2002). Multisensor fusion and integration: Approaches, applications, and future research directions. IEEE Sens. J..

[31] Subbarao M., Lu M. (1994). Image sensing model and computer simulation for CCD camera systems. Mach. Vis. Appl..

[32] Yun J., Choi T. Accurate 3-D shape recovery using curved window focus measure.

[33] Malik A., Choi T. (2007). Consideration of illumination effects and optimization of window size for accurate calculation of depth map for 3D shape recovery. Pattern Recognit..

[34] Shim S., Choi T. (2010). A novel iterative shape from focus algorithm based on combinatorial optimization. Pattern Recognit..

[35] Minhas R., Mohammed A.A., Wu Q.J. (2012). An efficient algorithm for focus measure computation in constant time. IEEE Trans. Circuits Sys. Video Technol..

